# The Role of Interfacial Adhesion in Polymer Composites Engineered from Lignocellulosic Agricultural Waste

**DOI:** 10.3390/polym13183099

**Published:** 2021-09-14

**Authors:** Dávid Kun, Zoltán Kárpáti, Erika Fekete, János Móczó

**Affiliations:** 1 Laboratory of Plastics and Rubber Technology, Department of Physical Chemistry and Materials Science, Budapest University of Technology and Economics, P.O. Box 91, H-1521 Budapest, Hungary; kun.david@mail.bme.hu (D.K.); karpatizol@gmail.com (Z.K.); bodine.fekete.erika@vbk.bme.hu (E.F.); 2 Institute of Materials and Environmental Chemistry, Research Centre for Natural Sciences, Eötvös Lóránd Research Network, P.O. Box 286, H-1519 Budapest, Hungary

**Keywords:** particle-reinforcement, adhesion, stress transfer, mechanical testing

## Abstract

This paper presents a comprehensive study about the application of a lignocellulosic agricultural waste, sunflower husk in different polymer composites. Two types of milled sunflower husk with different geometrical factors were incorporated into polypropylene, low-density and high-density polyethylene, polystyrene (PS), glycol-modified polyethylene terephthalate (PETG) and polylactic acid (PLA). The filler content of the composites varied between 0 and 60 vol%. The components were homogenized in an internal mixer and plates were compression molded for testing. The Lewis–Nielsen model was fitted to the moduli of each composite series, and it was found that the physical contact of the filler particles is a limiting factor of composite modulus. Interfacial interactions were estimated from two independent approaches. Firstly, the extent of reinforcement was determined from the composition dependence of tensile strength. Secondly, the reversible work of adhesion was calculated from the surface energies of the components. As only weak van der Waals interactions develop in the interphase of polyolefins and sunflower husk particles, adhesion is weak in their composites resulting in poor reinforcement. Interfacial adhesion enhanced by specific interactions in the interphase, such as π electron interactions for PS, hydrogen bonds for PLA, and both for PETG based composites.

## 1. Introduction

In recent years, sustainability has become a principle in many areas, including polymer science and engineering. As a result, more and more effort has been made to decrease the amount of fossil-based polymers and replace them with renewable, natural ones. However, these intentions are surrounded by a number of challenges since the processability and properties of natural polymers are inferior to those of petroleum-based plastics. Several approaches can be followed to eliminate these drawbacks, among which the preparation of polymer composites and blends is a relatively simple, efficient and economical option.

In the literature, numerous papers can be found about the utilization of bio-based as well as renewable, natural polymers in blends and composites. Bio-polyethylene [1,2], starch [3,4,5,6], protein [7,8] and lignin [9,10,11,12,13,14] have been used as a matrix or dispersed component of blends, while cellulose [15,16,17,18], chitin [5,19] and lignocellulose [4,9,20,21,22,23,24] have been applied as a reinforcement in composites. In addition, the advantageous properties of bio-based polymers and natural fibers were combined in their composites in several cases [1,18,24]. Among lignocellulosic fillers, many types of harvest wastes have been studied as well, including rice hulls [25], wheat straw [26], sugarcane bagasse [27], corn cobs [28], sunflower stalks [29], etc.

Sunflower husk is also a lignocellulosic waste material, and similarly to the above mentioned harvest wastes, it is available at low price and in large quantities. It consists of 34 wt% cellulose, 25 wt% lignin, 27 wt% hemicellulose and 13 wt% extractives [30]. Due to its considerable heat of combustion, it is used mostly as fuel to provide heat for the sunflower oil extraction process [31]. This means that the utilization of sunflower husk in composites could involve both economic and environmental advantages; however, this latter is somewhat reduced by the difficult handling of composites when they also become waste. In the paper of Angellier-Coussy et al. [32], several approaches are discussed for the waste management of lignocellulosic composites. Among them, a promising recycling procedure is shown by Grozdanov et al. [33]. The first step was the incorporation of rice husks and kenaf fibers into PLA, which was followed by the milling of these biocomposites. Eventually, the milled material was successfully applied as reinforcement in polyester resin.

Polymer/lignocellulose composites are used as structural materials in packaging [34,35], automotive industry [35,36,37], building and construction [35,38,39,40] and furniture production [35,38]. Polymer/sunflower husk composites could be applied for the same purposes. Nevertheless, only a few articles have been published about the application of sunflower husk as filler in polymer composites [41,42,43,44].

In the work of Sui et al. [41], polypropylene (PP) was filled with 5 wt% sunflower hull sanding dust (SHSD). According to the images taken by scanning electron microscope (SEM), the particles of SHSD were slightly fibrous with sizes in the range of 1–10 μm. Mechanical testing showed that the flexural modulus and strength of the composite were higher than those of the neat PP. The reinforcing effect of SHDS can be related probably to its slightly fibrous structure since the adhesion is poor between polyolefins and lignocelluloses due to their low surface energy [45]. The role of structure was also shown by Salasinska and Ryszkowska [42]. In their work, composites were prepared through the combination of finely ground sunflower husk and high-density polyethylene (HDPE) in a relatively wide composition range, from 5 to 30 wt% filler content. The modulus increased monotonically with increasing sunflower husk content, while tensile strength had a maximum at 15 wt% filler loading. Improved strength implies adequate stress transfer between the components which resulted from the high aspect ratio and the fine dispersion of the sunflower husk particles.

Marhoon [43] used sunflower husk as reinforcement in polyurethane composites. Sunflower husk was milled, sieved into different size fractions (<53, <75 and <106 μm) and added to flexible polyurethane until 10 wt% in 2 wt% steps. The modulus of elasticity and tensile strength increased with increasing filler content. Additionally, the gradient of the tendencies increased with decreasing particle size since smaller filler particles created larger contact surface area with the matrix polymer, which provided better stress transfer between the components. In another work, Barczewski et al. [44] prepared epoxy-based composites containing 15–35 wt% sunflower hull with an average particle size of 110.6 μm and an aspect ratio of 3.04. Tensile and flexural strength deteriorated upon the addition of sunflower husk, which were explained by the occurrence of voids during sample preparation as well as by the release of fat residues from the filler during the exothermic cross-linking process resulting in the plasticization of the composite.

The properties of polymer composites are affected by component characteristics, composition, structure and interfacial interactions. In many papers about composites, the primary focus is put on studying the role of structure while quite few articles discuss the role of interactions, and even fewer do that with quantitative analyses. In our previous paper [46], we investigated the role of interfacial adhesion in the composites of polyolefins and milled sunflower husk. At the interface between the components, interactions were modified by maleic-anhydride-grafted polyolefin coupling agents. The results unambiguously proved that coupling improved interfacial adhesion, which changed the dominant micromechanical deformation process from debonding to particle fracture. As a result, the strength of the composites increased considerably; however, their elongation-at-break values remained low, which may hinder application in practice.

For the engineering of interfacial interactions in composites, another simple approach is actually the selection of a matrix polymer with adequate surface properties. Although there are numerous articles about lignocellulose reinforced composites, in most cases only one or two types of polymers are used as a matrix component. This means that quantitative analysis is limited and general conclusions about the role of interactions can hardly be drawn. Therefore, we selected several thermoplastic polymers having different moduli, different surface energies, and that are capable of forming different intermolecular interactions with the applied filler. We filled these polymers with milled sunflower husk in a wide composition range to study quantitatively the effect of interfacial adhesion on the mechanical properties of the composites. In the experiments, we used two types of sunflower husk filler with different size and aspect ratio to investigate the possible role of structure, as well.

## 2. Experimental

### 2.1. Materials

Six commercially available thermoplastic polymer grades were used as matrix, namely polypropylene (PP), low-density polyethylene (LDPE), high-density polyethylene (HDPE), polystyrene (PS), glycol-modified polyethylene terephthalate (PETG) and polylactic acid (PLA). All of these polymers were used in the form of pellets with 1–2 mm diameter. The type, source and the most important properties of the polymers used in this study are provided in Table 1. Two types of milled sunflower husk were applied as filler in the composites, and both of them were supplied by Bunge (Chesterfield, Missouri, USA). The SunPro Fiber (SPF) and SunPro 20 (SP20) grades are already milled and they contain slightly fibrous particles. The average particle length of SPF is 2600 µm, the aspect ratio is 3.3, and the density is 1.42 g/cm^3^, while these characteristics of SP20 are 1100 µm, 2.8, and 1.44 g/cm^3^.

### 2.2. Sample Preparation

Prior to sample preparation, sunflower husk fillers were dried in the air at 105 °C for 12 h in a ventilated oven, while PETG and PLA were kept at 200 mbar air pressure and 105 °C for 4 h in a vacuum oven to remove their humidity content. The components were homogenized in a Brabender W 50 EHT internal mixer at 42 cm^3^ charge volume and 50 rpm. First, the polymer was melted within 1–2 min, then the filler was added and mixing was carried out for additional 10 min. Set temperature was 190 °C for the PS and PETG composites, 180 °C for the PP and PLA ones, as well as 160 °C for the LDPE and HDPE ones. The filler content of the composites increased from 0 to 30 vol% in 5 vol% steps, and from 30 to 60 vol% in 10 vol% steps.

Immediately after mixing, 1-mm-thick plates were compression molded from the still plastic materials using a Fontijne SRA 100 machine. The temperature of compression molding was set at the same value as that of the internal mixer for each material. After the plates had been stored at room temperature and 50% relative humidity for one week, 5 tensile bars (type 1A ISO) were machined from each composite for further testing.

### 2.3. Characterization

The surface tension of unmilled sunflower husk and the polymers applied was determined by static contact angle measurements using the OWRK method [47,48,49,50]. Diiodomethane was used for the determination of the dispersion component of surface tension, while water, ethylene glycol and formamide were applied for the estimation of the polar component. The contact angle of 20 μL liquid droplets was measured at 23 °C and 50% relative humidity with a Ramé–Hart goniometer.

Mechanical properties (modulus, strength and elongation-at-break) were determined by tensile testing using an Instron 5566 universal testing machine. Gauge length was 115 mm and the cross-head speed was set at 5 mm/min. The structure of the composites was studied by digital optical microscopy (DOM) using a Keyence VHX 5000 apparatus. Micrographs were recorded on the compressed surface of the plates.

### 2.4. Statistical Analysis

The modulus of the sunflower husk particles was estimated by applying the model of Lewis and Nielsen [51]. The model was fitted to the moduli of the composites by nonlinear regression using the Generalized Reduced Gradient Nonlinear algorithm. The iteration steps were done by the Solver add-in of Microsoft Excel (Version 2016, Microsoft, Redmond, WA, USA).

Analysis of covariance (ANCOVA) was performed to determine the statistical significance of structure and interfacial adhesion in the reinforcing effect of sunflower husk. The level of significance was set at 0.05, thus a factor was considered to be significant in case its *p*-value was smaller than 0.05. Calculations were carried out by means of Statistica software (Version 13.3, TIBCO Software, Palo Alto, CA, USA).

## 3. Results and Discussion

The results are presented and discussed in several sections. Firstly, the factors limiting the modulus of the composites are studied. Secondly, the composition dependence of strength and elongation-at-break is presented, which expresses the performance of the composites at failure. Thirdly, the reinforcing effect of sunflower husk is analyzed quantitatively, and eventually, it is related to interfacial adhesion and structure.

### 3.1. The Limiting Factors of Modulus

**Figure 1:**
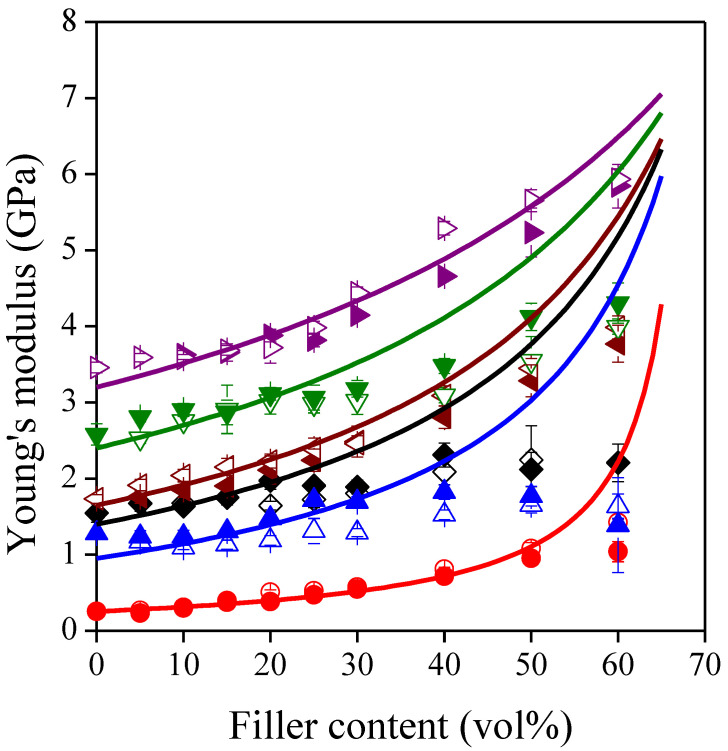
Effect of filler content on the stiffness of the composites. Symbols: (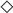) PP; (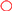) LDPE; (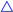) HDPE; (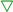) PS; (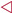) PETG; (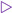) PLA; empty symbols: SP20; full symbols: SPF; Lewis–Nielsen model.

A number of applications are subjected to static loading; thus, they must be engineered with adequate stiffness to maintain their dimensions. In many cases, the modulus of neat polymers is too low, thus their particulate filled composites are used instead. Many papers have shown that the incorporation of lignocellulosic fillers can enhance the modulus of polymers [4,9,20,21,22,23,24]; however, the limiting factors are rarely discussed. In Figure 1, the modulus of the composites is plotted as a function of sunflower husk content. Young’s modulus increases with increasing filler content for all the composite series studied since the lignocellulosic particles of sunflower husk have a higher modulus than the polymer matrices. For a better understanding of the tendencies, the semiempirical model of Lewis and Nielsen [38] was fitted to the measured moduli by nonlinear regression. This model can be expressed by the following equations.

(1) $$E=E_{m}\frac{1+AB\varphi }{1-B_{E}\psi \varphi }$$

(2) $$A=\frac{7-5\nu_{m}}{8-10\nu_{m}}$$

(3) $$B_{E}=\frac{\frac{E_{f}}{E_{m}}-1}{\frac{E_{f}}{E_{m}}+A}$$

(4) $$\psi =1+\frac{1-\varphi_{max}}{\varphi_{max}^{2}}\varphi$$

where *E*, *E_m_* and *E_f_* are the Young’s moduli {GPa} of the composite, the matrix and the filler, respectively, *ν_m_* is the Poisson’s ratio {mm/mm} of the matrix, *φ* is the filler content {cm^3^/cm^3^}, and *φ_max_* is the maximum packing fraction {cm^3^/cm^3^} of the filler. The two parameters, *A* and *Ψ*, are related to the structure of the composite; however, they are not very well defined [52]. Parameter *A* can be related to filler anisotropy, through the relation *A* = *k_E_* − 1, where *k_E_* is the Einstein’s coefficient, but the relation has not been thoroughly investigated and verified yet. Parameter *Ψ* is the function of the maximum packing fraction, thus it is related to anisotropy, but it is affected also by the formation of an interphase. Despite these uncertainties, the Lewis–Nielsen model is quite frequently used to predict the modulus of particulate filled composites [52]. In order to estimate *φ_max_*, the sunflower husk particles were assumed to be ellipsoids having aspect ratios between 2.8 and 3.3, and being in maximally random jammed state. Based on the simulations of Donev et al. [53], the maximum packing density is approximately 0.67 for such particles, therefore we used this value as *φ_max_*.

**Figure 2:**
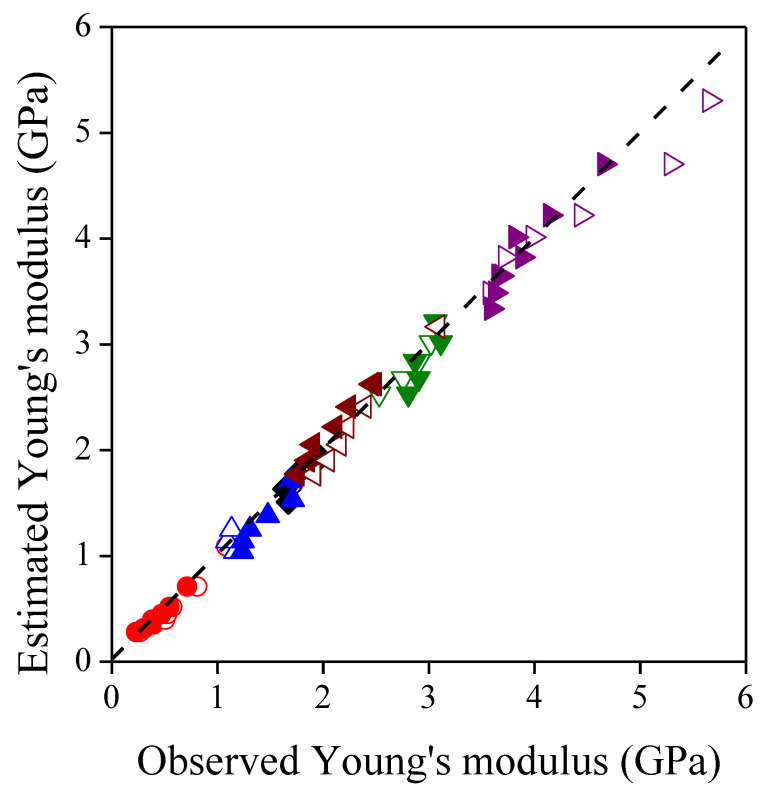
Estimated moduli as a function of observed moduli representing the goodness-of-fit for the Lewis–Nielsen model. Symbols: (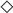) PP; (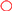) LDPE; (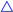) HDPE; (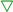) PS; (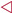) PETG; (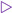) PLA; empty symbols: SP20; full symbols: SPF.

The average modulus of the filler particles was an output of the nonlinear regression, thus its value determined the fitted curves. When all moduli were involved in the nonlinear regression, the Lewis–Nielsen model did not fit adequately to the data. The deviation between the observed and fitted data was the highest at large filler contents, which indicates the appearance of a factor being neglected by the model. The validity range was determined by removing the observed moduli one by one from 60 vol% to lower filler contents, and then by re-fitting the model to the remained data. For both fillers, the results of the best fits are summarized in Table 2, while the goodness-of-fit is demonstrated by Figure 2 showing the estimated moduli plotted against the observed moduli from the validity interval. The modulus of the two filler types does not differ significantly from each other, therefore the Lewis–Nielsen model was re-fitted to the data of all the composite series studied, and the fitted curves were also placed in Figure 1. The common modulus of sunflower husk particles was found to be 7.50 ± 0.71 GPa, which is close to the modulus of lignocellulosic filler materials with similar size, anisotropy and composition [54,55,56], but it is inferior compared to those with a more fibrous structure and higher cellulose content [56].

**Figure 3:**
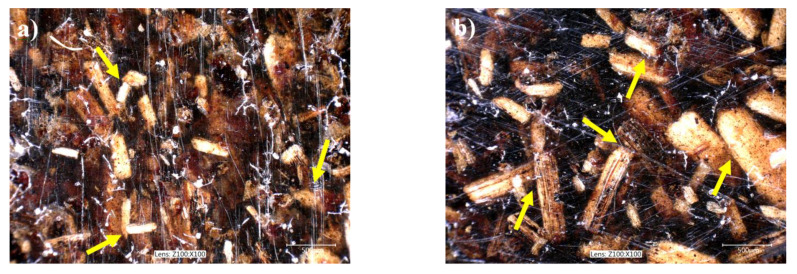
Physical contact between the sunflower husk particles in HDPE composites. Arrows show some physical contact points. The white scratches are the imprints of the steel mold. Filler content: 40 vol%. (**a**) SP20; (**b**) SPF.

The limited validity of the Lewis–Nielsen model can be attributed to the surface properties of the components. Firstly, we can assume poor interfacial adhesion between the milled sunflower husk and the polymers possessing low surface energy, such as poliolefins and PS, which could result in the debonding of matrix/filler interface around larger particles even at very small deformations where the modulus of the composites was determined [57,58]. Secondly, we can also assume that the wettability of the sunflower husk is poor, which results in the physical contact of their particles at higher filler contents as shown by the DOM images of Figure 3. Since these associations are held together only by weak interactions, they can be easily disrupted [57,59]. This effect can induce the formation of voids around the filler particles resulting in lower modulus than expected.

### 3.2. Performance at Failure

**Figure 4:**
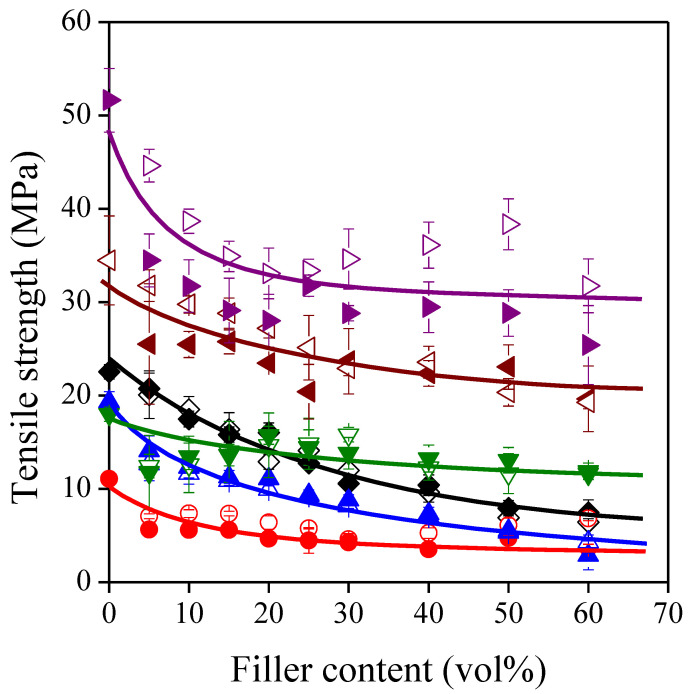
Effect of filler content on the strength of the composites. Symbols: (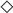) PP; (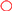) LDPE; (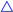) HDPE; (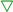) PS; (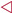) PETG; (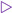) PLA; empty symbols: SP20; full symbols: SPF.

The practical relevance of a composite material is demonstrated not only by its modulus but also by the mechanical properties measured at failure, such as strength and deformation-at-break. In Figure 4, tensile strength is plotted against sunflower husk content. In all cases, strength decreases with increasing filler loading, but the gradient of the tendencies is quite different. On an absolute scale, the strength of PLA based composites decreases drastically, while that of LDPE and PS based ones changes slightly. The decrease of strength is often considered to be a consequence of weak interfacial adhesion between the matrix polymer and the filler. However, strength is affected not only by interfacial interactions but also by matrix [60] and filler properties [61], as well as by structure [62], thus a proper analysis of the tendencies requires the application of adequate models (see next section).

**Figure 5:**
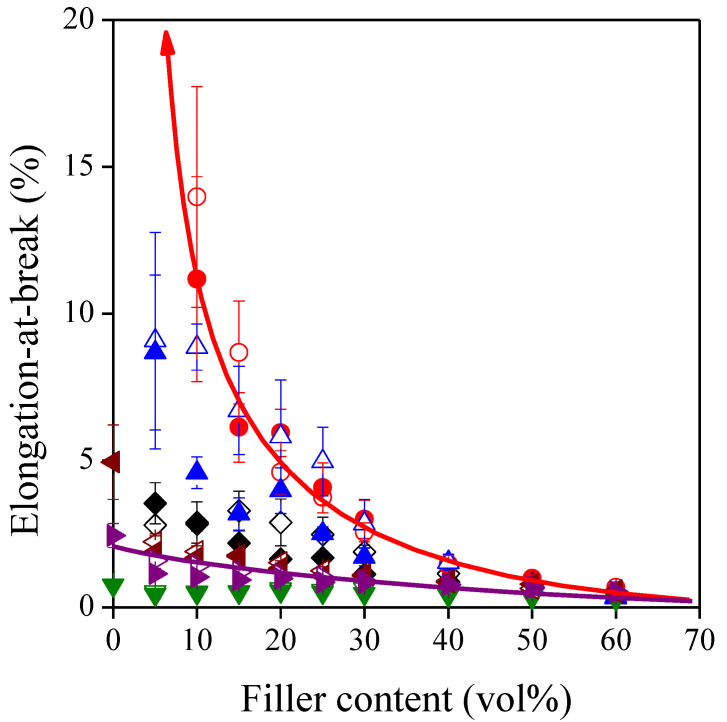
Effect of filler content on the brittleness of the composites. Symbols: (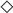) PP; (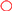) LDPE; (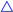) HDPE; (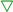) PS; (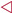) PETG; (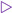) PLA; empty symbols: SP20; full symbols: SPF. One trend line represents the LDPE composites having the highest deformability, and the other one represents the much more rigid PLA composites.

**Figure 6:**
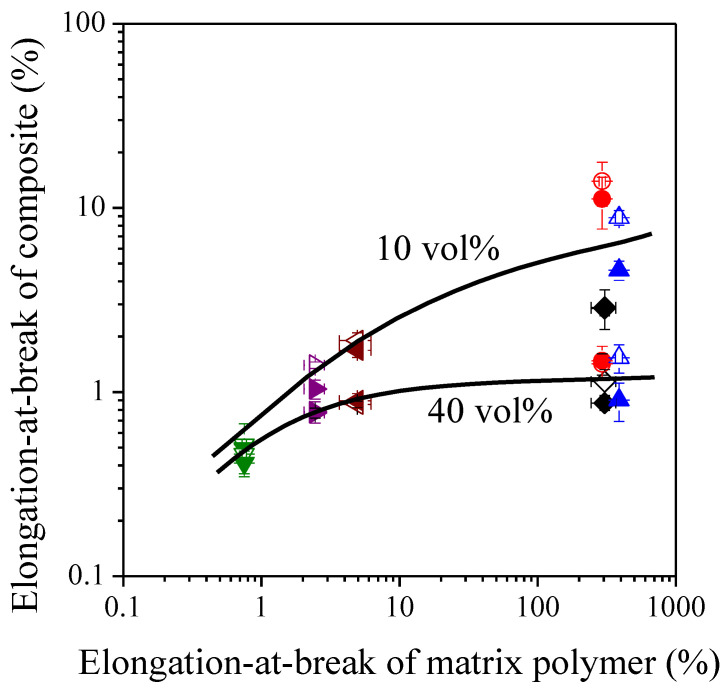
Correlation between the deformability of the matrix polymer and that of their composites with 10 vol% and 40 vol% filler content, respectively. Symbols: (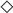) PP; (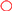) LDPE; (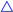) HDPE; (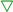) PS; (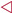) PETG; (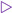) PLA; empty symbols: SP20; full symbols: SPF.

The brittleness of the composites studied is well demonstrated by their elongation-at-break values which are presented as a function of sunflower husk content in Figure 5. Deformability is decreased significantly by the incorporation of the rigid filler particles results. The highest elongation-at-break values were measured for the polyolefin based composites, while the lowest values were determined for the PS based systems. This observation implies that the tendencies are mostly determined by the deformability of the matrix polymer, which is proved by Figure 6 showing the correlation between the elongation-at-break of the matrix polymer and that of the composites at 10 vol% and 40 vol% filler loading, respectively. The data are plotted on a logarithmic scale since they cover a range of 3–4 orders of magnitude.

### 3.3. Reinforcing Effect of Filler

As was mentioned earlier, the strength of a composite is affected by many factors including interfacial adhesion, matrix and filler properties, as well as structure [60,61,62]. The application of the model proposed by Pukánszky et al. [63] offers the possibility to study these factors quantitatively. This semi-empirical model is based on the Nicolais–Narkis model [64], but it uses an effective load-bearing cross-section of the matrix, as well as it takes into account the influence of interfacial interactions. The composition dependence of tensile strength is expressed by the following formula [63].

(5) $$\sigma_{Tred}=\sigma_{T}\frac{1+2.5\varphi }{1-\varphi }\frac{1}{\lambda^{n}}=\sigma_{Tm}\exp\left(B\;\varphi \right)$$

where *σ_Tred_* is the reduced tensile strength {MPa}, *σ_T_* and *σ_T__m_* are the true tensile strength {MPa} of the composite and the matrix, respectively (*σ_T_* = *σλ* and *λ* = *L*/*L*_0_, where *L* is the ultimate and *L*_0_ the initial gauge length {mm} of the specimen), *n* is a parameter taking into account strain hardening {dimensionless}, *φ* is the volume fraction {cm^3^/cm^3^} of the filler, and *B* is related to its relative load-bearing capacity {dimensionless}, i.e., to the extent of reinforcement. Parameter *B* is determined by the size of the interface between the matrix and the filler and by the properties of the interphase [65]

(6) $$B=\left(1+A_{d}\;\rho_{d}\;\ell \right)\ln\left(\frac{\sigma_{i}}{\sigma_{T0}}\right)$$

where *A_d_* and *ρ_d_* are the specific surface area and density of the filler, while *ℓ* and *σ_i_* are the thickness of the interphase and its strength, respectively. Since the thickness of the interphase (*ℓ*) depends on the strength of interactions, parameter *B* can provide information also about interfacial adhesion.

**Figure 7:**
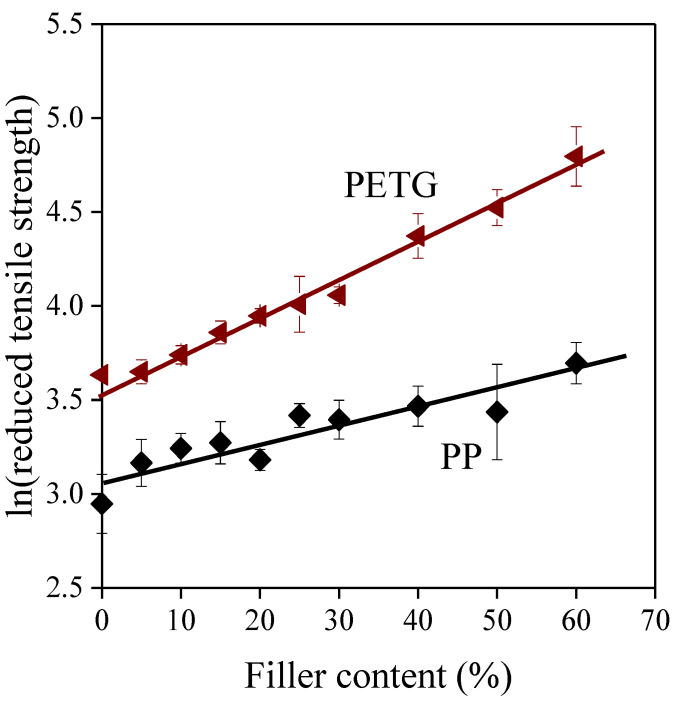
Reduced tensile strength of two composite series plotted against filler content in the form of Equation (7). Symbols: (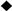)) PP/SP20; (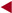)) PETG/SP20. Empty symbols represent data omitted from fitting.

If we take the logarithm of the two sides of Equation (5), we receive the following linear form

(7) $$ln\sigma_{Tred}=ln\left(\sigma_{T}\frac{1+2.5\varphi }{1-\varphi }\frac{1}{\lambda^{n}}\right)=ln\sigma_{Tm}+B\;\varphi$$

where the dependent variable is the natural logarithm of reduced tensile strength, the independent variable is the volume fraction of the filler, the intercept is the natural logarithm of the matrix strength, and the slope is equal to parameter *B.* The reduced tensile strength of PP/SP20 and PETG/SP20 composite series is plotted this way with the fitted linear curves in Figure 7, while the fitting results are summarized in Table 3 for all the series studied. Based on the fitted *B* values, two major groups can be distinguished. For the poliolefin based composites, parameter *B* is quite small implying the presence of only weak van der Waals forces in the interphase. The other composites have higher *B* values, which can originate from the formation of specific interactions in the interphase, such as π electron interactions for PS [66,67,68,69], hydrogen bonds for PLA [70], and both for PETG based systems [66,67,68,69,70].

**Figure 8:**
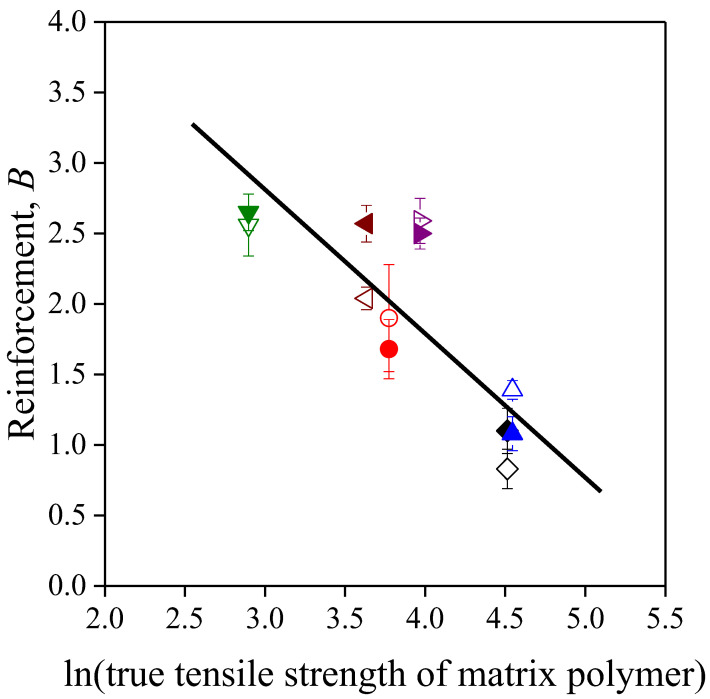
Correlation between reinforcement and matrix strength in the composites studied. Symbols: (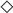) PP; (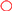) LDPE; (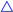) HDPE; (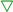) PS; (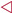) PETG; (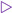) PLA; empty symbols: SP20; full symbols: SPF.

According to Equation (6), there is a linear correlation between parameter *B* and the logarithm of matrix strength, which is corroborated by Figure 8. This implies that *B* values contain not only the effect of interfacial adhesion but also that of matrix properties, and more reliable conclusions could be drawn from the *B* values if they were made independent of the matrix properties. For this purpose, we used a correction proposed in our previous paper [71] to give a more accurate estimation of reinforcement, thus parameter *B* was multiplied by the natural logarithm of the true tensile strength of the matrix polymer (*B*ln*σ_Tm_*). These values are also listed in Table 3, and, indeed, they show a slightly different picture of reinforcement. The lowest *B*ln*σ_Tm_* values, i.e., the smallest extent of reinforcement, still belong to the polyolefin based composites. The reinforcing effect of filler particles is increased somewhat in the PS matrix as π electron interactions may develop in the interphase. If hydrogen bonds can develop between the components, the extent of reinforcement increases further. As a result, the reinforcing effect of the lignocellulosic fillers can be utilized mostly in PETG and PLA. This statement contradicts somewhat the considerable decreasing tendencies of strength for these two polymers (Figure 4). A possible explanation is that we can expect low inherent strength for the sunflower husk particles applied due to their disadvantageous geometrical factors [61].

### 3.4. Effect of Adhesion and Structure on Reinforcement

**Figure 9:**
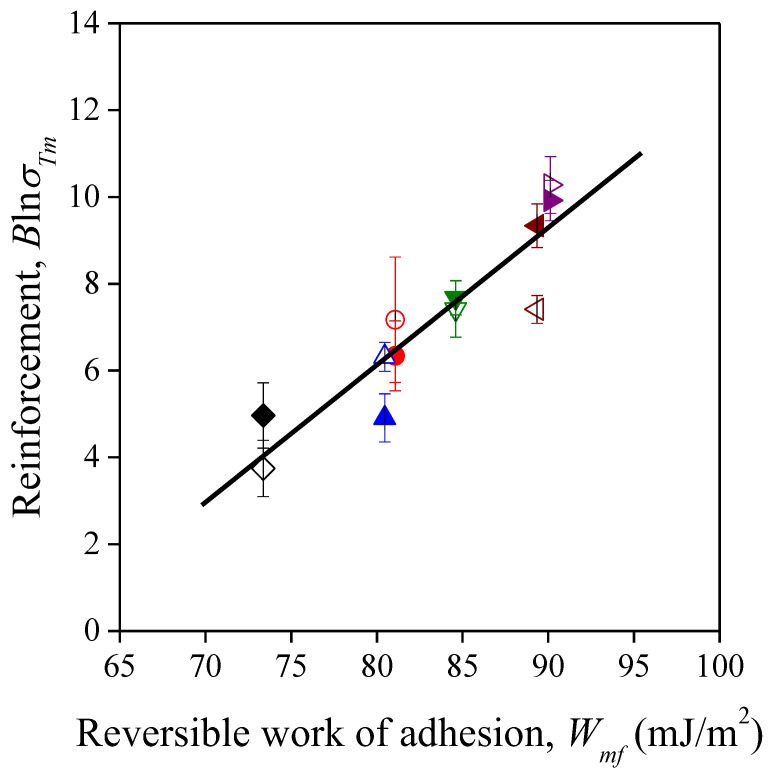
Correlation between reversible work of adhesion and reinforcement in the composites studied. Symbols: (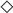) PP; (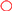) LDPE; (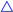) HDPE; (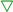) PS; (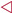) PETG; (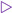) PLA; empty symbols: SP20; full symbols: SPF.

Another approach for the estimation of interfacial adhesion is the determination of the reversible work of adhesion. In this work, the reversible work of adhesion was calculated from the surface tension of the components. According to the theoretical assumptions of Fowkes [72], the surface free energy is the sum of contributions from the different intermolecular forces at the surface, thus the surface tension can be divided into a dispersion and a polar component. This latter includes also specific forces, such as π electron interactions and hydrogen bonds. As a result, the reversible work of adhesion can be estimated also by the following formula [73]

(8) $$W_{mf}=2\sqrt{\gamma_{m}^{d}\cdot \gamma_{f}^{d}}+2\sqrt{\gamma_{m}^{p}\cdot \gamma_{f}^{p}}$$

where *W_mf_* is the reversible work of adhesion between the matrix and the filler, *γ* is the surface tension, the subscripts *m* and *f* represent the matrix and the filler, respectively, while the subscripts *d* and *p* denote the dispersion and polar component of surface tension, respectively. The dispersion and polar surface tension of sunflower husk was found to be 41.3 mJ/m^2^ and 1.8 mJ/m^2^, respectively, while the calculated *W_mf_* values with the measured *γ* values of the polymers are collected in Table 4. We can draw the same conclusions from these results as from the *B*ln*σ_Tm_* values shown in Table 3, and even the order of the data is the same. This observation is visualized by the linear correlation between them in Figure 9.

The results of statistical analysis, namely ANCOVA, also corroborated the dominant role of adhesion in the determination of reinforcement. The effect of the reversible work of adhesion was statistically significant (*p* = 0.0028), while that of the filler type was not (*p* = 0.8912). Although the particle size was quite different in the two types of milled sunflower husk used in our experiments (1100 µm for SP20, and 2600 µm for SPF), our previous results about lignocellulosic composites showed that the aspect ratio of a filler particle is a more relevant factor than particle size [62]. Therefore, the negligible effect of filler structure may be related to the similar aspect ratio of the lignocellulosic fillers (2.8 for SP20, and 3.3 for SPF).

## 4. Conclusions

In this paper, a comprehensive study is presented about the role of interfacial adhesion and structure in polymer/lignocellulose composites. The lignocellulosic filler was sunflower husk which is an agricultural waste available at low price and in large quantities. The modulus of the composites is limited by several factors. In polymer matrices with low surface energy, the debonding of matrix/filler interface around larger particles may occur even at very small deformations. In addition, at larger filler contents, sunflower husk particles physically contact each other resulting in very weak associations. Interfacial adhesion was estimated quantitatively from the extent of reinforcement and the reversible work of adhesion. Both approaches provided concordant results about interfacial interactions. Only weak van der Waals forces act in the interphase of polyolefin based composites, which results in poor adhesion and reinforcement. For the PS based systems, the reinforcing effect of sunflower husk particles is increased, which can be related to by the formation of π electron interactions in the interphase. Among the polymers studied, interfacial adhesion is the strongest in the PLA and PETG based composites since hydrogen bonds can also develop in the interphase. Regarding filler structure, no difference was found between the reinforcing effects of the two types of milled sunflower husk used in our experiments, which can be explained by their similar aspect ratio. For all series, stiff and rigid composites are obtained at large sunflower husk loadings, which could be mitigated by the application of elastomers. The relatively low strength of the composites might be improved by increasing the inherent strength of sunflower husk particles. For this purpose, both the modification of filler geometry by further milling and the chemical treatment of the filler particles could be beneficial.

## Figures and Tables

**Table 1:** The most important properties of the matrix polymers used in the experiments.

Polymer	Trade Name	Producer	Density (g/cm^3^)	MFR ^a^ (g/10 min)	Temp. (°C) and Load (kg) of MFR
PP	H 649 FH	MOL Group (Budapest, Hungary)	0.90	2.5	230, 2.16
LDPE	FA-244-51		0.92	0.28	190, 2.16
HDPE	Tipelin 7100S		0.95	0.25	190, 5.00
PS	Styron 686 E	Americas Styrenics (The Woodlands, TX, USA)	1.05	2.5	200, 5.00
PETG	Ecozen SE	SK Chemicals (Seongnam, Korea)	1.27	10.9	250, 2.16
PLA	Ingeo 4032	NatureWorks (Minnetonka, MN, USA)	1.24	3.9	190, 2.16

^a^ Melt flow rate.

**Table 2:** Fitting results of the Lewis–Nielsen model [38].

Filler	Upper Composition Limit of Model Validity (vol% Filler Content)						Filler Modulus ^a^ (GPa)	R^2 b^
	PP	LDPE	HDPE	PS	PETG	PLA		
SP20	0.15	0.50	0.15	0.20	0.40	0.50	7.63 ± 0.45	0.8859
SPF	0.20	0.40	0.30	0.25	0.30	0.40	7.45 ± 0.69	0.8519

^a^ Error shows the 95% confidence interval; ^b^ coefficient of determination indicating the goodness of fit.

**Table 3:** Reinforcing effect of sunflower hull in the polymers studied.

Matrix Polymer	Filler	True Tensile Strength of Matrix Polymer, *σ_Tm_* (MPa)	*B*	R^2 a^	*B*ln*σ_Tm_*
PP	SP20	91.19 ± 17.04	0.83 ± 0.14	0.8339	3.75
	SPF		1.10 ± 0.16	0.8731	4.96
LDPE	SP20	43.56 ± 4.08	1.90 ± 0.38	0.8299	7.17
	SPF		1.68 ± 0.21	0.9274	6.34
HDPE	SP20	94.20 ± 8.90	1.39 ± 0.07	0.9882	6.32
	SPF		1.08 ± 0.12	0.9456	4.91
PS	SP20	18.13 ± 0.78	2.56 ± 0.22	0.9558	7.42
	SPF		2.65 ± 0.13	0.9847	7.68
PETG	SP20	37.83 ± 2.54	2.04 ± 0.08	0.9895	7.41
	SPF		2.57 ± 0.13	0.9834	9.34
PLA	SP20	52.88 ± 3.40	2.59 ± 0.16	0.9748	10.28
	SPF		2.50 ± 0.11	0.9877	9.92

^a^ Determination coefficient indicating the goodness of the fit.

**Table 4:** The dispersion and polar surface tension components of the polymers used in the study, and the reversible work of adhesion in their composites.

Matrix Polymer	Surface Tension (mJ/m^2^)		Reversible Work of Adhesion, *W_mf_* (mJ/m^2^)
	Dispersion Component, *γ_S_^d^*	Polar Component, *γ_S_^p^*	
PP	39.0	0.2	73.4
LDPE	35.3	3.1	81.1
HDPE	37.2	0.6	80.5
PS	40.5	1.1	84.6
PETG	43.7	2.7	89.3
PLA	43.2	4.5	90.1

## Data Availability

The data presented in this study are available on request from the corresponding author.
